# A comprehensive genomic analysis provides insights on the high environmental adaptability of *Acinetobacter* strains

**DOI:** 10.3389/fmicb.2023.1177951

**Published:** 2023-04-17

**Authors:** Yang Zhao, Hua-Mei Wei, Jia-Li Yuan, Lian Xu, Ji-Quan Sun

**Affiliations:** ^1^Lab for Microbial Resources, School of Ecology and Environment, Inner Mongolia University, Hohhot, China; ^2^Jiangsu Key Lab for Organic Solid Waste Utilization, Educational Ministry Engineering Center of Resource-Saving Fertilizers, Jiangsu Collaborative Innovation Center for Solid Organic Waste Resource Utilization, Nanjing Agricultural University, Nanjing, China

**Keywords:** *Acinetobacter*, comparative genomic analysis, environmental adaptability, horizontal gene transfer (HGT), metabolic

## Abstract

*Acinetobacter* is ubiquitous, and it has a high species diversity and a complex evolutionary pattern. To elucidate the mechanism of its high ability to adapt to various environment, 312 genomes of *Acinetobacter* strains were analyzed using the phylogenomic and comparative genomics methods. It was revealed that the *Acinetobacter* genus has an open pan-genome and strong genome plasticity. The pan-genome consists of 47,500 genes, with 818 shared by all the genomes of *Acinetobacter*, while 22,291 are unique genes. Although *Acinetobacter* strains do not have a complete glycolytic pathway to directly utilize glucose as carbon source, most of them harbored the *n*-alkane-degrading genes *alkB*/*alkM* (97.1% of tested strains) and *almA* (96.7% of tested strains), which were responsible for medium-and long-chain *n*-alkane terminal oxidation reaction, respectively. Most *Acinetobacter* strains also have *catA* (93.3% of tested strains) and *benAB* (92.0% of tested strains) genes that can degrade the aromatic compounds catechol and benzoic acid, respectively. These abilities enable the *Acinetobacter* strains to easily obtain carbon and energy sources from their environment for survival. The *Acinetobacter* strains can manage osmotic pressure by accumulating potassium and compatible solutes, including betaine, mannitol, trehalose, glutamic acid, and proline. They respond to oxidative stress by synthesizing superoxide dismutase, catalase, disulfide isomerase, and methionine sulfoxide reductase that repair the damage caused by reactive oxygen species. In addition, most *Acinetobacter* strains contain many efflux pump genes and resistance genes to manage antibiotic stress and can synthesize a variety of secondary metabolites, including arylpolyene, β-lactone and siderophores among others, to adapt to their environment. These genes enable *Acinetobacter* strains to survive extreme stresses. The genome of each *Acinetobacter* strain contained different numbers of prophages (0–12) and genomic islands (GIs) (6–70), and genes related to antibiotic resistance were found in the GIs. The phylogenetic analysis revealed that the *alkM* and *almA* genes have a similar evolutionary position with the core genome, indicating that they may have been acquired by vertical gene transfer from their ancestor, while *catA*, *benA*, *benB* and the antibiotic resistance genes could have been acquired by horizontal gene transfer from the other organisms.

## Introduction

Members of the genus *Acinetobacter*, which is in the family Moraxellaceae and order Gamma-proteobacteria, are ubiquitous in various natural environments (Al Atrouni et al., 2016; Adewoyin and Okoh, 2018), including soil, oceans, freshwater, sediments, activated sludge, and sites contaminated with hydrocarbons (Carr et al., 2003; Rocha et al., 2013; Krizova et al., 2014; Zhang et al., 2020). For example, the relative abundances of *Acinetobacter* in a municipal dumpsite, forest air, and Rarh’s laterite soil were 12, 20.9, and 31%, respectively (Mwaikono et al., 2016; Fang et al., 2020; Mukhopadhyay et al., 2021). In some environments, such as drinking water and water contaminated with crude oil under microaerophilic conditions, the abundances of *Acinetobacter* can be as high as 47.5 and 66.3%, respectively (Van Assche et al., 2019; Revesz et al., 2020). Previous studies have already determined that the *Acinetobacter* species have multiple metabolic capabilities and biological activities, such as drug resistance (Kyriakidis et al., 2021), the production of antioxidants (Qadir et al., 2021), immunosuppressive activities (Kim et al., 2017), and the degradation of sulfamethoxazole (SMX; Wang and Wang, 2018), herbicides (Dong et al., 2015), organophosphorus pesticides (Liu et al., 2007), alkanes (Sun et al., 2012), aromatic compounds (Thangaraj et al., 2008), natural or synthetic polymers, and other widespread environmental pollutants (Rajoo et al., 2013). This suggests that strains of *Acinetobacter* are abundant in their genetic, ecological, and physiological diversity. Furthermore, some species of the *Acinetobacter* are infamous drug-resistant bacteria that are common pathogens that cause nosocomial human infection. They include *A. baumannii*, *A. pitti*, *A. lwoffii*, and *A. nosocomialis* (Shin and Park, 2017).

The *Acinetobacter* species are considered to be model microorganisms in the field of environmental microbiology for their ecological and clinical importance (Metzgar et al., 2004; Jacobs et al., 2014). Currently, a substantial amount of research on *Acinetobacter* species has focused on the identification of antibiotic resistance mechanisms (Godeux et al., 2022), diagnosis of pathogens (Shan et al., 2022), genome analysis and evolution (Jia et al., 2022), gene plasticity (Vijayakumar et al., 2022), and horizontal gene transfer (HGT) (Mindlin et al., 2021). A comparison of the genomes of 16 *A. johnsonii* strains revealed that the clinically derived strains accumulated more functional genes related to translation modification, β-lactamase biosynthesis, and defense mechanisms, and the strains derived from the environmental usually accumulated more functional genes related to the degradation of compounds (Jia et al., 2022). The resistance profiles of *A. johnsonii* were found to be generated by crossing or the co-selection of anthropogenic contaminants and mediated by efflux pumps instead of the corresponding resistance determinants (Jia et al., 2021). A comparative analysis of the genomes of 163 *Acinetobacter* strains showed that the acquisition of specific virulence factors could contribute to the broad persistence and virulence of *A. baumannii* (Sahl et al., 2013). However, there has been less systematic information on the environmental adaptability of *Acinetobacter* strains. Therefore, in this study, the phylogenomic and comparative genomics of 312 strains of *Acinetobacter* were analyzed to elucidate the potential mechanisms of niche adaptation and stress tolerance.

## Materials and methods

### Genome source and analysis of *Acinetobacter* strains

As of March 2021, there were 1,631 *Acinetobacter* strains with published genome sequences in the GenBank. The qualities of the downloaded 1,631 genome sequences were verified using CheckM (Parks et al., 2015). To ensure the accuracy of the analysis, only 312 genomes with completion >99% and contamination <1% were selected for further phylogenomic and comparative genomic analyses. More detailed information on the 312 *Acinetobacter* genomes is listed in Supplementary Table S1. Among them, 207 strains were isolated from clinical-related habitats, 32 strains from animal-related habitats, and 73 strains from environmental habitats. The clinical strains include those isolated from places related to hospitals, such as hospital sewage, urine, blood, sputum, wounds, and ears among others. The animal strains were isolated from animal meats and animal feces. Environmental strains refer to strains isolated from the natural environment, including soil, sludge, and water among others.

*Acinetobacter* currently includes 74 species with valid published names, but 90 of the genomes used in this study have not been assigned to a specific species of *Acinetobacter*. To elucidate the accurate taxonomic position of the unassigned *Acinetobacter* strains, the average nucleotide identity based on BLAST (ANIb) values were calculated using the online ANI calculator^1^ (Richter et al., 2016). The digital DNA–DNA hybridization (dDDH) values were calculated using the Genome to Genome Distance Calculator (GGDC 2.5)^2^ (Auch et al., 2010). An ANIb value above 95% for two organisms and a dDDH value above 70% indicate that they belong to the same species (Chun et al., 2018).

### Pan-genome analysis of *Acinetobacter*

The genome sequences were annotated using the Prodigal. The pan-and core-genome analyses of the *Acinetobacter* genome were conducted by the Bacterial Pan Genome Analysis tool (BPGA) pipeline v. 1.3 with the default parameters (Chaudhari et al., 2016). In the BPGA pipeline, orthologous protein clusters were identified with USEARCH using a threshold of 0.5. The pan genome curve is generated by plotting the total number of distinct gene families against the number of genomes considered. Similarly, the number of shared gene families is plotted against the number of genomes to produce a core genome plot. In a pan-genome analysis, the number of accumulated genes related to the number of genomes can be expected by Heaps’ law (Tettelin et al., 2008):

(1) $$\;f{\left(x\right)}_{pan}=a.x^{b}$$

where x is the number of genomes; $f{\left(x\right)}_{pan}$ is the size of pan-genome, and a and b are fitting parameters. Based on the Heaps’ law, the pan-genome is open when 0 < b < 1, while b < 0 indicates a closed pan-genome. The exponential curve fitting model of the core genome data is as follows:

(2) $$f{\left(x\right)}_{core}=c.e^{-d.x}$$

where $f{\left(x\right)}_{core}$ is the size of core genome, and c and d are fitting parameters.

### COG and the analysis of main metabolism

The Clusters of Orthologous Groups of proteins (COG) database through BPGA (v. 1.3) was used to categorize the core, accessory, and unique gene families. To analyze the accuracy and completeness of major metabolic signatures, the metabolic profiles predicted from the Kyoto Encyclopedia of Genes and Genomes (KEGG) pathway were assigned to genes to identify the major metabolic signatures among the 21 complete genomes of *Acinetobacter* strains.

### Search for functional genes in the genomes

The genes related to osmotic stress, oxidative stress, and hydrocarbon degradation were searched in 21 complete genomes, and the sequences obtained were compared with the reference sequences in GenBank to ensure that they were correct. We subsequently used these sequences as reference sequences and used the BLAST tool in NCBI^3^ to analyze the remaining 312 genomes. Sequence with the best match and > 40% identity to the sequence in the database were selected. The obtained sequences were then aligned using the CLUSTAL_X tool and corrected manually. They were used for the phylogenetic and evolutionary analyses.

### Phylogenetic analysis

The phylogenomic tree based on the core genes was constructed using BPGA with default settings. In addition, the Interactive Tree Of Life online server (iTOL)^4^ was used to display the phylogenetic tree (Letunic and Bork, 2021). The amino sequences of the medium-chain alkane hydroxylase (AlkB/AlkM), long-chain alkane hydroxylase (AlmA), benzoate 1,2-dioxygenase (BenAB) and catechol 1,2-dioxygenase (CatA) were aligned using ClustalW (Hall, 2013). A phylogenetic tree was constructed using the neighbor-joining algorithm (Saitou and Nei, 1987) in MEGA software v. 6.0 (Tamura et al., 2013). The tree topology was evaluated using the bootstrap analysis based on 1,000 resampling replicates.

### Identification of the genes for secondary metabolism and antibiotic resistance

The secondary metabolite biosynthetic gene clusters (smBGCs) were predicted using the antiSMASH v. 6.0 online^5^ with the default settings (Blin et al., 2021). Because the genes from the same cluster can be scattered across multiple contigs, the prediction of smBGCs using the draft or contig-level genomes often increases the number of clusters predicted. Therefore, only 21 complete *Acinetobacter* genomes were used to ensure the accuracy of smBCGs predictions (Lee et al., 2020). The antibiotic resistant genes were identified by searching using the resistance gene identifier (RGI) search of the CARD^6^ with the protein homology model (Alcock et al., 2020). The sequence with the Perfect, Strict and Loose hits identities >70% were selected for further analysis.

### Analysis of the HGT events

Horizontal gene transfer (HGT) involves the rapid introduction of newly evolved genes into the existing bacteria, which enables them to rapidly adapt to the external environment (Jain et al., 2003). The genes acquired by HGT can be predicted by comparing the G + C content and phylogenetic differences between candidate genes and the whole genome and analyzing flanking mobile genetic elements (Syvanen, 1994; Garcia-Vallve et al., 2000). However, the “gold standard” for the identification of HGT is phylogenetic discordance, manifested by significant conflict between the gene trees and species trees (Keeling and Palmer, 2008).

Integrated prophages were identified using the PHAST server^7^ (Arndt et al., 2016). The genomic islands (GIs) in these genomes were identified using the IslandViewer 4 server^8^ (Bertelli et al., 2017), which integrates four methods to predict the GIs, including IslandPick (Langille et al., 2008), SIGI-HMM (Waack et al., 2006), IslandPath-DIMOB (Hsiao et al., 2003), and Islander (Hudson et al., 2015).

## Results

### Basic information about the *Acinetobacter* strains

The genome size of the 312 *Acinetobacter* strains ranged from 2.41 to 4.76 Mb, while the genomic DNA G + C content ranged from 34.9 to 45.4%. The number of coding sequences (CDS) ranged from 2,173 to 4,399. The largest genome and CDS numbers were found in *Acinetobacter* sp. Ac_5812, which was isolated from lettuce (*Lactuca sativa*), while the smallest was *A. apis* ANC 5114, which was isolated from honeybee (*Apis mellifera*) guts. In addition, strains with the highest and lowest amounts of genomic DNA G + C content were *A. indicus* CIP 110367^T^, which was isolated from a constructed environment, and *A. equi* 114^T^, which was isolated from horse dung. Several *Acinetobacter* species can be found in clinical, animal and environmentally relevant habitats, respectively, such as *A. pittii*, *A. calcoaceticus*, *A. radioresistens*, *A. lwoffii*, *A. junii*, *A. terrae*, *A. soli* and *A. oleivorans*.

A total of 33 strains that were not assigned to species were identified using a genomic analysis (Table 1). Among them, strains EKM10A and AKBS16 were assigned to *A. baumannii*; strains ANC 5045, YH1901147 and YT-02 were assigned to *A. seohaensis*, and strains 1239920, 1461402, and 230853 were assigned to *A. radioresistens*. In addition, five, five, and six strains were identified as *A. pittii* (strains TG27347, 907131, TG2027, BEC1-S18-ESBL-01, and BS1), *A. lwoffii* (strains ANC 5347, NIPH 713, YH18001, ANC 5324, and 18QD2AZ41W) and *A. nosocomialis* (strains 1179249, 1424608, FDAARGOS_541, 1130196, 1245593, and TUM15103), respectively. Strains 1437282 and 146457 had been identified as *A. baumannii*, but according to our calculations, they should be *A. courvalinii* (ANIb values 96.2 and 96.1% and dDDH values 71.0 and 70.3%, respectively).

**Table 1:** Tentative taxonomic assignment of the unnamed *Acinetobacter* genus identified in this study.

Taxon	Reference strain	Closest species or taxon	ANIb (%)	dDDH (%)
*Acinetobacter* sp. EKM10A	ATCC 19606	*Acinetobacter baumannii*	97.39	80.30
*Acinetobacter* sp. AKBS16			97.49	80.80
*Acinetobacter* sp. 1179249	NIPH 2119	*Acinetobacter nosocomialis*	97.61	82.20
*Acinetobacter* sp. 1424608			97.56	81.30
*Acinetobacter* sp. FDAARGOS_541			97.61	81.70
*Acinetobacter* sp. 1130196			97.49	80.60
*Acinetobacter* sp. 1245593			97.7	83.00
*Acinetobacter* sp. TUM15103			97.84	83.40
*Acinetobacter* sp. TG27347	DSM 30006	*Acinetobacter pittii*	96.44	71.50
*Acinetobacter* sp. 907131			96.62	72.60
*Acinetobacter* sp. TG2027			96.37	71.30
*Acinetobacter* sp. BEC1-S18-ESBL-01			96.75	73.00
*Acinetobacter* sp. BS1			96.82	73.90
*Acinetobacter* sp. NIPH 3623	CCM 8635	*Acinetobacter courvalinii*	99.96	99.90
*Acinetobacter baumannii* 1437282			96.19	71.00
*Acinetobacter baumannii* 146457			96.13	70.30
*Acinetobacter* sp. 1239920	DSM 6976	*Acinetobacter radioresistens*	97.84	86.20
*Acinetobacter* sp. 1461402			98.99	95.30
*Acinetobacter* sp. 230853			99.01	95.20
*Acinetobacter* sp. ANC 5045	DSM 16313	*Acinetobacter seohaensis*	96.83	77.50
*Acinetobacter* sp. YH1901147			96.67	78.30
*Acinetobacter* sp. YT-02			96.46	74.10
*Acinetobacter* sp. ANC 5347	NCTC 5866	*Acinetobacter lwoffii*	96.44	73.50
*Acinetobacter* sp. NIPH 713			96.32	72.60
*Acinetobacter* sp. YH18001			96.42	72.40
*Acinetobacter* sp. ANC 5324			96.23	70.60
*Acinetobacter* sp. 18QD2AZ41W			96.22	71.20
*Acinetobacter* sp. NIPH 899	NIPH 2171	*Acinetobacter variabilis*	96.28	70.90
*Acinetobacter* sp. SFC	ANC 4282	*Acinetobacter terrae*	98.05	84.20
*Acinetobacter* sp. ANC 4973	ANC 5109	*Acinetobacter kyonggiensi*	97.75	81.60
*Acinetobacter* sp. ANC 4216	ANC 4667	*Acinetobacter kookii*	97.22	77.00
*Acinetobacter* sp. NIPH 542	DR1	*Acinetobacter oleivorans*	96.62	72.70
*Acinetobacter* sp. SK-43	CIP 64.5	*Acinetobacter junii*	96.87	80.00
*Acinetobacter* sp. CIP 102637	DSM 16617	*Acinetobacter parvus*	96.27	72.60
*Acinetobacter* sp. 243_ASPC	KCTC 22184	*Acinetobacter soli*	98.51	88.70

**Figure 1:**
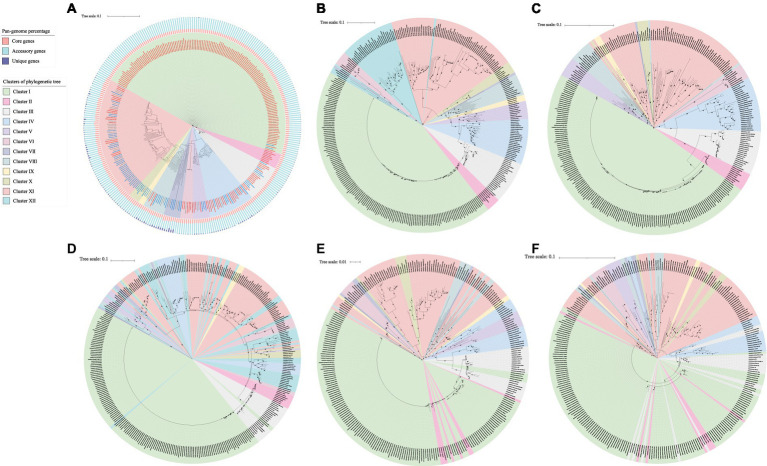
Phylogenetic tree of *Acinetobacter* constructed based on the amino acid sequences of core genes **(A)**, AlkM **(B)**, AlmA **(C)**, CatA **(D)**, BenA **(E)**, and BenB **(F)**. Clinical strains are indicated in red font; animal strains are indicated in gray font, and environmental strains are indicated in blue font. The three colors in the bar graph represent the percentage of core, accessory, and unique genes in the pan-genome, respectively.

The phylogenetic tree based on the core genome of *Acinetobacter* suggested that these 312 strains could be assigned to 11 clusters (Figure 1A). The clinical strains were primarily distributed in clusters I, II, IV, and VI. The strains in cluster I were all *A. baumannii*. Representative strains in clusters II and VI included *A. nosocomialis* and *A. radioresistens*, respectively, and those in cluster IV were *A. beijerinckii* and *A. junii*. The animal and environmental strains were primarily distributed in clusters VII, VIII, IX, X and XI. Among them, cluster IX is composed of *A. kanungonis*, and cluster X is composed of *A. indicus* and *A. seohaensis*, while clusters VII, VIII and XI are composed of many different species of *Acinetobacter*. Cluster VII consists of *A. brisouii*, *A. larvae*, *A. pullicarnis*, *A. marinus*, *A. nectaris*, *A. baretiae*, *A. apis* and *A. pollinis*. *A. gerneri*, *A. lanii*, *A. shaoyimingii*, *A. chinensis*, *A. rongchengensis*, *A. piscicola* and *A. wuhouensis* formed cluster VIII. Cluster XI includes *A. kookii*, *A. harbinensis*, *A. albensis*, *A. kyonggiensi*, *A. terrae*, *A. terrestris*, *A. johnsonii*, *A. equi*, *A. portensis*, *A. schindleri*, *A. celticus*, *A. bouvetii*, *A. cumulans*, *A. tianfuensis*, *A. gandensis*, *A. variabilis*, *A. idrijaensis*, and *A. lwoffii*.

### Pan-genome and COG distribution analysis

The pan-genome defines the entire genomic repertoire of a given phylogenetic clade and encodes for all possible lifestyles carried out by its organisms (Vernikos et al., 2015). The pan-genome contains the core genome, accessory genomes, and strain-unique genes. Among them, the core genome is essential for the basic lifestyle of bacteria, while the accessory genome provides species diversity, environmental adaptability and other characteristics (Tettelin et al., 2008).

**Figure 2:**
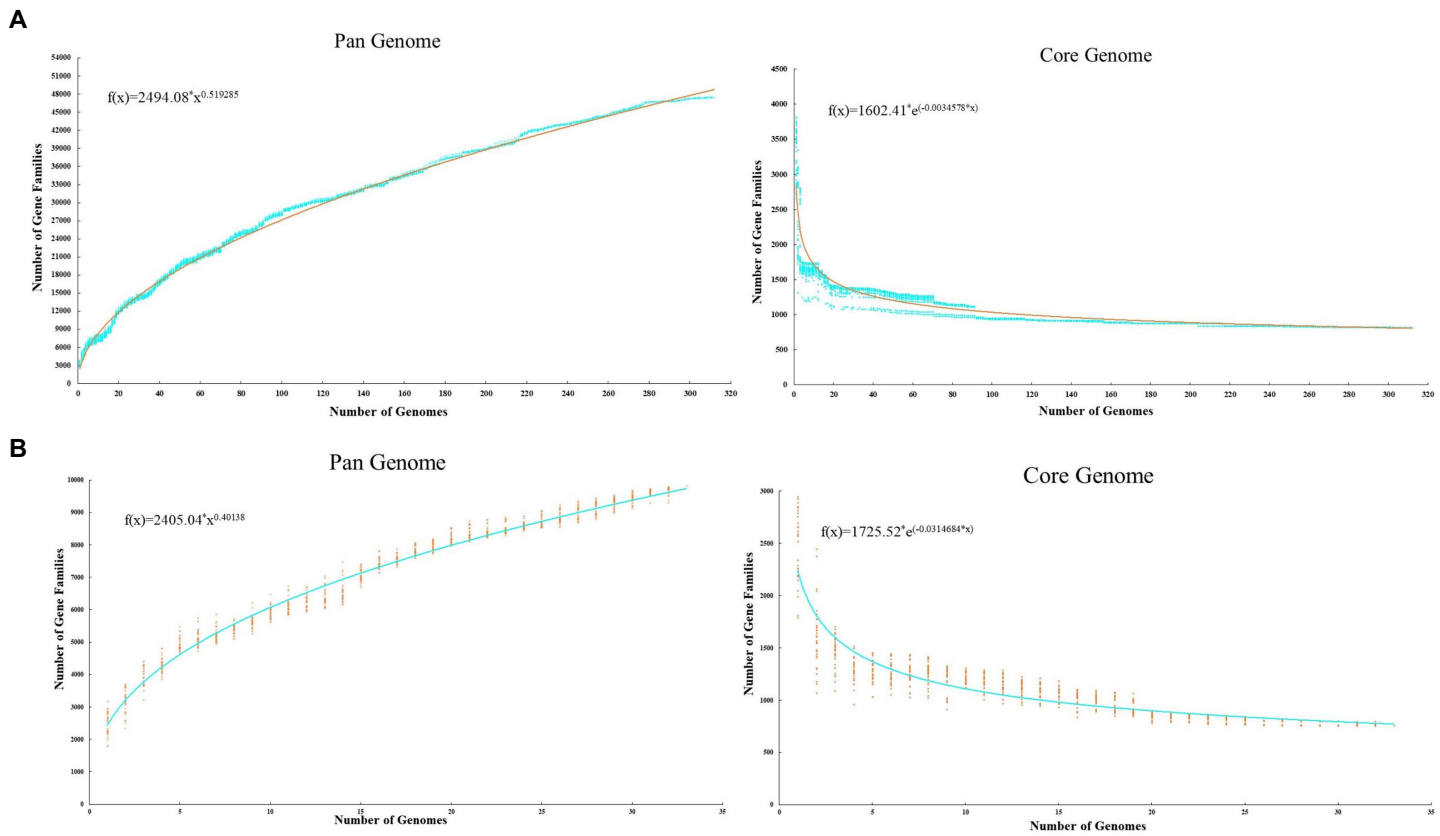
Pan and core genome plot of 312 *Acinetobacter* strains **(A)** and 33 typic *Acinetobacter* strains **(B)**. The plot shows how the number of gene families increase and decline in the pan and core genome with each consecutive addition of the *Acinetobacter* genome.

To take a comprehensive view of the *Acinetobacter* genome and further explore the genomic diversity of this genus, we calculated the size of the pan-genome based on different datasets. When all 312 *Acinetobacter* genomes were analyzed, the results showed that the pan-genome contained 47,500 gene families, of which the numbers of core, accessory and unique genes were 818, 24,391, and 22,291, respectively. The genomes of 312 *Acinetobacter* isolates were primarily composed of the core genes (19.8–37.8%) and accessory genes (44.6–78.7%), with about more than half of the accessory genes indicating that *Acinetobacter* has high environmental adaptability. The number of unique genes in the strain ranged from 0 to 700, and the high numbers of unique genes indicated the significant differences between the *Acinetobacter* genomes. When three strain genomes were selected from each cluster of the core genome phylogenetic tree (11 clusters), and analyzed based on functional genes (removed hypothetical protein and unknown protein genes) only, a total of 758 core genes, 5,089 accessory genes, and 3,953 unique genes were identified in the pan-genome. Similarly, these genomes were composed mainly of core genes (23.8–42.6%), accessory genes (41.5–72.6%) and contained different numbers of unique genes (18–508). According to Heaps’ law, the pan-genome of 312 (*b* = 0.52) and 33 (*b* = 0.40) *Acinetobacter* genomes were both open, indicating that with each newly added genome, the number of new genes increases the genetic repertoire of the species (Figure 2).

The COG analysis revealed that most categories in the 312 *Acinetobacter* core (39.0%) and accessory (31.3%) genomes were related to metabolism, while the unique genomes were related to information storage and processing (35.4%) (Supplementary Figure S1A). In the core genome, class J (translation, ribosomal structure, and biogenesis) was the most abundant cluster (14.5%), while class Q (secondary metabolite biosynthesis and transport and catabolism) was the least abundant (1.4%). In addition, class E (9.8%; amino acid transport and metabolism), class F (5.0%; nucleotide transport and metabolism), class H (6.8%; coenzyme transport and metabolism), class C (5.7%; energy production and conversion), and class O (5.3%; post-translational modification, protein turnover, and chaperones) related to the maintenance of primary cellular processes were also abundant in the core genome (Supplementary Figure S1B). Compared with the core genome, the accessory genomes were enriched the most in class G (carbohydrate transport and metabolism), class Q, and class P (inorganic ion transport and metabolism) with percentages of 6.5% (4.3%), 4.0% (2.2%) and 4.0% (1.4%), respectively. The unique genes were most enriched in class U (intracellular transport, secretion, and vesicular transport), class K (transcription), class V (defense mechanisms) and class Q, and their percentages were 8.9% (2.3%), 10.0% (5.3%), 3.4% (0.7%) and 3.3% (1.4%), respectively. Similarly, the core genome of 33 *Acinetobacter* strains was most enriched in classes J, E, F, H, C and O, the accessory genome in classes G, Q and P, and the unique genes in classes U, K, V and Q (Supplementary Figure S2). This suggests that the accessory genomes and unique genes are different from the core genome, and are mainly related to environmental niche adaptation functions, such as transportation and metabolism of substances and defense mechanism.

### Analysis of the main metabolism and secondary metabolites in *Acinetobacter* strains with complete genomes

**Figure 3:**
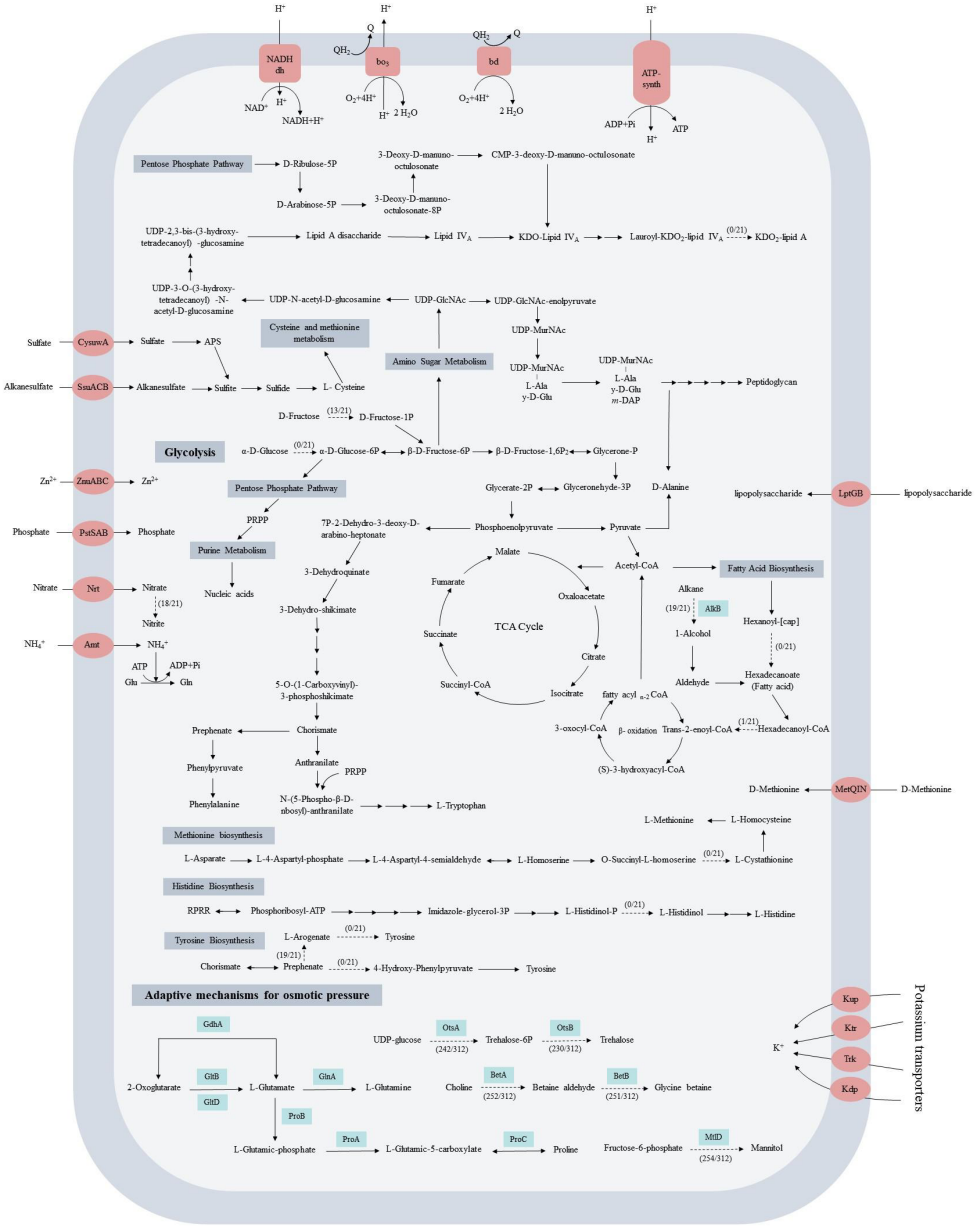
Prediction of the central metabolic potential of 21 complete genomes of *Acinetobacter* strains and the possible hyperosmolar mechanisms of adaptation of 312 strains. Potential genes related to carbon, nitrogen, and energy metabolism in the central metabolic pathways were analyzed, and the genes predicted to be involved in these metabolic pathways are listed in Supplementary Table S2. Solid lines indicate pathways common to all bacteria, and dashed lines indicate partial presence.

The KEGG analysis revealed that all 21 strains harbored the intact gluconeogenesis pathway, pentose phosphate pathway, tricarboxylic acid cycle, purine and pyrimidine synthesis, fatty acid and peptidoglycan synthesis pathway, and the incomplete glycolytic pathway (Figure 3; Supplementary Table S2). None of the 21 *Acinetobacter* strains could convert glucose to glucose-6-P because they lacked the hexokinase (HK) gene, and this gene was not found in the other 291 strains too. In terms of nitrogen metabolism, all 21 strains could take up ammonia using the ammonium transport (Amt) family of ammonium transporters. Four strains were unable to take up and utilize nitrate directly because they lacked the complete nitrate reduction pathway. Among them, *A. equi* 114^T^ has nitrate reductase but lacks nitrite reductase, while *A. larvae* BRTC-1^T^, *A. wanghuae* dk386 and *A. chinensis* WCHAc010005^T^ have neither. The remaining 17 strains have assimilatory nitrate reductase (NarB) and dissimilatory nitrite reductase (NirBD), which can convert nitrate to ammonia. In terms of sulfur metabolism, all 21 strains have a complete sulfate reduction pathway. In addition, the core genome harbored a set of phosphate, sulfate, alkanesulfate, zinc, lipopolysaccharide, and D-methionine absorption proteins, which enable *Acinetobacter* strains to obtain and utilize nutrients such as carbon, nitrogen, phosphorus and sulfur from the environment to ensure its survival in different environments.

Amino acids are essential for metabolism and catabolism in living cells. The genome analysis indicated that 21 *Acinetobacter* strains could synthesize 16 types of amino acids, including alanine, aspartate, glutamate, glutamine, glycine, serine, threonine, cysteine, valine, isoleucine, leucine, lysine, arginine, proline, phenylalanine, and tryptophan. However, they could not synthesize methionine, histidine, tyrosine, and asparagine. Τhe lack of cystathionine gamma-synthase precluded the synthesis of methionine; histidinol-phosphatase precluded the synthesis of histidine; prephenate dehydrogenase precluded the synthesis of tyrosine, and asparagine synthase precluded the synthesis of asparagine. Except for the histidinol-phosphatase gene was found in *Acinetobacter* sp. FDAARGOS 515, these four enzymes were not found in other 291 strains. In addition, the genes for lipopolysaccharide biosynthesis, vitamin metabolism, peroxidase, and resistance to β-lactam and cationic antimicrobial peptide (CAMP) were also found in the core genomes, which could endow the *Acinetobacter* strains with the ability to more effectively adapt to a complex and changeable environment.

**Figure 4:**
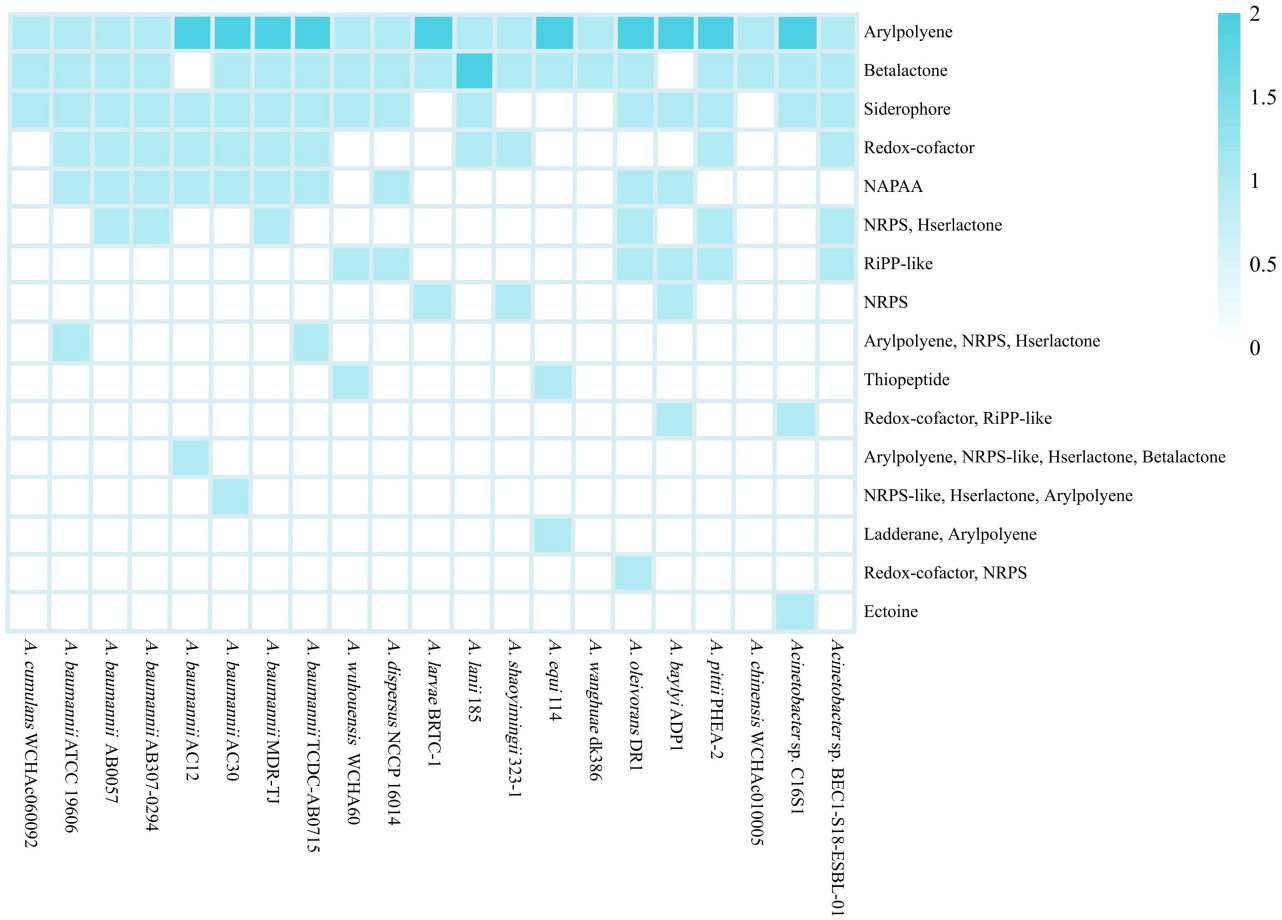
Distribution of the smBGCs in 21 *Acinetobacter* complete genomes. NAPAA, non-alpha poly-amino acids; NRPS, non-ribosomal peptide synthetase cluster; RiPP, ribosomally synthesized and post-translationally modified peptide product cluster.

Secondary metabolites are types of organic molecules that are not required for basic survival. However, they often have diverse and powerful biological functions by which the host strains are able to adapt to the environment (Abegaz and Kinfe, 2019). The genes that encode the enzymes involved in the synthesis of secondary metabolites are often clustered into biosynthetic gene clusters (BGCs; Medema et al., 2015). The *Acinetobacter* strains usually harbored two to eight secondary metabolite biosynthetic gene clusters (smBGCs) per genome. A total of 114 smBGCs of 12 major classes were predicted in 21 genomes, and six were more than 50% similar to the known clusters; 63 were less than 30% similar, and 34 had no similarity (Supplementary Table S3). In addition, a small number of smBGCs were heterozygous clusters, which refers to clusters that harbor more than one type of metabolite gene, and 14 heterozygous clusters were found in 13 strains. *A. oleivorans* DR1^T^ harbored the highest numbers of smBGCs, while strains *A. wanghuae* dk386 and *A. chinensis* WCHAc010005^T^ each harbored only two smBGCs. In detail, the smBGCs responsible for the biosynthesis of aryl polyenes (APEs) were found in all 21 strains (Figure 4). The exact role of APE is currently unclear, but the frequent presence of APE BGCs in commensal and pathogenic bacteria has led to hypotheses that its primary function is evasion of the host immune system (Lee et al., 2021a,b). The smBGCs related to β-lactone and siderophore biosynthesis were found in 19 and 16 strains, respectively. Two putative types of siderophore clusters were detected in 16 strains. A total of 13 clusters were similar to the acinetoferrin cluster (three with 70% similarity, one with 40% similarity and nine with 30% similarity) and three clusters were similar to the staphylobactin cluster (three with 12% similarity). Only three of these were highly similar to known BGCs, suggesting that there could be new types of siderophores in *Acinetobacter*. In addition, some smBGCs are strain-specific, which may be related to the external environment of the strains. For example, the BGC responsible for ectoine synthesis was only annotated in *Acinetobacter* sp. C16S1, and the BGC for the biosynthesis of ladderane was only detected in *A. equi* 114^T^.

### Distribution of the genes for the degradation of alkane and aromatic hydrocarbon in the *Acinetobacter* genomes

Alkanes and aromatic hydrocarbons are the two primary types of chemicals that are widely distributed in nature. For example, cuticular wax in the epidermal cuticle of plant leaves is a complex mixture of linear C_20_-C_60_ aliphatic compounds (Samuels et al., 2008). Insects can biosynthesize pheromones with a hydrocarbon backbone (Tillman et al., 1999). Cyanobacteria can synthesize C_15_ to C_19_ hydrocarbons and release them into the environment (Lea-Smith et al., 2015). Anthropogenic inputs are often considered to be the primary source of polycyclic aromatic hydrocarbons (PAHs) in the natural environment. They are usually introduced by oil spills and incomplete combustion among others (Jiang et al., 2007). In addition, many microorganisms can degrade alkanes and aromatic hydrocarbons, and these two hydrocarbons can be used as carbon and energy sources for strains to grow.

**Figure 5:**
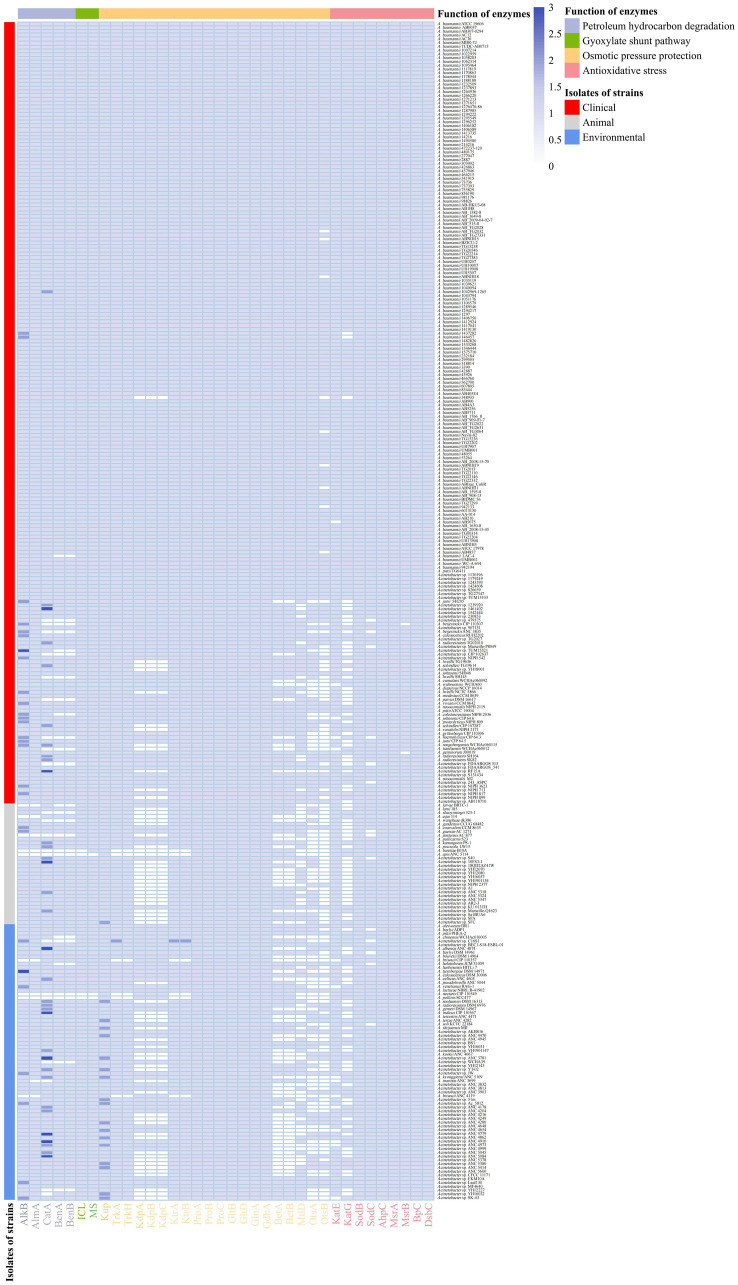
Distribution of enzymes with functions of petroleum hydrocarbon degradation, osmotic pressure protection and anti-oxidative stress in 312 strains. Petroleum hydrocarbon degrading enzymes, AlkM, AlmA, CatA, BenA, BenB; Glyoxylate shunt pathway enzymes, ICL, MS; Potassium ion transporters, Kup, TrkA, TrkH, KdpA, KdpB, KdpC KtrA, KtrB; Compatible solute synthases, ProA, ProB, ProC, GltB, GltD, GlnA, GdhA, BetA, BetB, MtlD, OtsA, OtsB; Antioxidant stress kinases, KatE, KatG, SodB, SodC, AhpC, MsrA, MsrB, BpC, DsbC.

Alkane hydroxylases, which catalyze the hydroxylation of alkanes, are key enzymes involved in the aerobic degradation of alkanes by bacteria. The primary alkane hydroxylases include the methane monooxygenase pMMO and butane monooxygenase BMO (Austin and Groves, 2011), the membrane-bound non-heme iron alkane monooxygenase AlkB/AlkM for the oxidation of medium-and long-chain alkanes (Van Beilen et al., 2006), and the two flavin-containing enzymes AlmA and LadA for the oxidation of long-chain alkanes (Throne-Holst et al., 2007). Only the *alkM* and *almA* genes were found in these *Acinetobacter* strains. Among them, 331 *alkM* genes were found in 303 strains, while 302 *almA* genes were found in 302 strains. A total of 302 *Acinetobacter* strains (approximately 96.8%) simultaneously have the *alkM* genes and the *almA* genes. Alternatively, the *alkM* gene was not found in nine strains, while the *almA* gene was not found in 10 strains. Nine strains that lack the *alkM* and *almA* genes include *A. apis* ANC 5114, *A. baretiae* B10A, *A. brisouii* CIP 110357^T^, *A. brisouii* ANC 4119, *A. equi* 114, *A. larvae* BRTC-1^T^, *A. nectaris* CIP 110549, *A. pollinis* SCC477, and *A. portensis* AC 877^T^. Strain *A. marinus* ANC 3699 isolated from sea water has the *alkM* gene but does not have the *almA* gene. A total of 26 strains contained two *alkM* genes, and two strains harbored three copies of the *alkM* genes (Figure 5; Supplementary Table S1).

Benzoic acid is converted to catechol catalyzed by benzoate 1,2-dioxygenase (Ben) under aerobic conditions (Collier et al., 1998). The resulting catechol is then degraded to *cis*, *cis*-muconate by catechol 1,2-dioxygenase (CatA) or degraded to 2-hydroxymuconic semialdehyde by catechol 2,3-dioxygenase (C23O; Oh et al., 1997; Wells and Ragauskas, 2012). Naphthalene dioxygenase (NDO) catalyzes the incorporation of two atoms of molecular oxygen into naphthalene to form *cis*-(1R,2S)-dihydroxy-1,2-dihydronaphthaleneor or the *cis*-dihydroxylation of biphenyl and phenanthrene (Parales et al., 2000). A total of 334 *catA* genes (in 291 strains) and 287 *benA* and *benB* genes (in 287 strains) were found in the *Acinetobacter* strains. Among them, 285 *Acinetobacter* strains (approximately 91.3%) simultaneously harbor the *catA*, *benA* and *benB* genes. *catA* was not found in 21 strains, and *benA* and *benB* were not found in 25 strains. A total of 19 strains did not harbor any *catA*, *benA,* and *benB* genes. Among them, nine, five, and five strains were isolated from clinical, animal, and environmental sources, respectively. *A. marinus* ANC 3699 was isolated from sea water, and *Acinetobacter* sp. YH16032 that was isolated from dust contained only the *benA* and *benB* genes, while *A. colistiniresistens* NIPH 2036^T^, *Acinetobacter* sp. TUM15521, *A. baumannii* LAC-4, *A larvae* BRTC-1^T^, *A. chinensis* WCHAc010005^T^, and *A. halotolerans* JCM 31009^T^ only harbored the *catA* gene. In addition, 25 strains harbored two *catA* genes, and nine strains harbored three copies of the *catA* gene (Figure 5). The C23O and NDO genes were not found in any of these *Acinetobacter* strains.

The glyoxylate shunt (GS) is a well-known TCA variant of alkane and fatty acid metabolism. GS is a carbon metabolism process from isocitrate to malate *via* glyoxylate, and is catalyzed by isocitrate lyase (ICL, encoded by the *aceA* gene) and malate synthase (MS, encoded by the *glcB* gene; Park et al., 2019). 308 strains had the GS process, and the four strains that did not have the GS pathway also lacked both alkane and aromatic hydrocarbon degradation genes (Figure 5). They were *A. baretiae* B10A^T^ and *A. apis* ANC 5114 isolated from honeybee gut, as well as *A. nectaris* CIP 110549^T^ and *A. portensis* AC 877^T^ isolated from nectar. In addition, only *A. portensis* AC 877^T^ isolated from pork lacked both alkane and aromatic hydrocarbon degradation genes. The genome analysis indicated that most *Acinetobacter* strains can degrade *n*-alkanes and aromatics as carbon sources for their growth.

### The ways in which *Acinetobacter* adapts to osmotic stress

When microorganisms are exposed to a high osmotic pressure environment, the water in the cells will flux out rapidly along the osmotic gradient, which may cause a reduction in turgor and the dehydration of the cytoplasm (Kempf and Bremer, 1998). Microorganisms usually adopt two primary strategies to alleviate intracellular and extracellular osmotic pressure (Figure 3). One is designated the “salt in the cytoplasm” strategy in which the strains usually accumulate a large amount of K^+^ in their cytoplasm (Epstein, 2003; Gunde-Cimerman et al., 2018). The accumulation of potassium in the cytoplasm relies on the K^+^ transporter. The Kdp, Trk, Kup and Ktr systems are the common K^+^ transporters in bacteria (Schloesser et al., 1993; Epstein, 2016). The Kdp system in bacteria has a strong affinity for potassium ions. The Trk system is often related to the pathogenicity of microorganisms (Valente and Xavier, 2016). The Kup and Ktr systems are transport systems with a low affinity for the potassium ion and serve as secondary potassium ion transport systems in most bacterial strains (Epstein, 2016). Genes related to the Kup system were found in the genomes of all 312 *Acinetobacter* strains, while the genes related to the Trk, Kdp, and Ktr systems were found in 306, 245, and 306 genomes of *Acinetobacter* strains, respectively (Figure 5). Most *Acinetobacter* strains have two to four K^+^ transport systems. In particular, *A. baretiae* B10A^T^ and *A. apis* ANC 5114, which were both isolated from honeybee guts, only have the Kup system. All the *Acinetobacter baumannii* strains except for *A. baumannii* 348935 have four K^+^ transport systems. In addition, all the clinical strains except for *A. baumannii* have three to four K^+^ transport systems, while only 11 strains had three K^+^ transport systems and lacked a Kdp system. Animal strains have three to four K^+^ transport systems, and 23 and 7 strains have 3 and 4 K^+^ transport systems, respectively, while the environmental strains have two to four K^+^ transport systems. Four, 29 and 40 strains have two, three, and four K^+^ transport systems, respectively.

Another strategy relies on the biosynthesis and accumulation of compatible organic solutes (Ventosa et al., 1998), such as sugars (e.g., trehalose), polyols (e.g., mannitol), amino acids (e.g., glutamate and proline) and their derivatives (e.g., ectoine and betaine). These compatible solutes not only maintain the osmotic balance of the cell, but also serve as stabilizers of proteins and cellular components, preventing denaturing effects of high ionic strength (Kempf and Bremer, 1998). Choline dehydrogenase (BetA) catalyzes the oxidation of choline to betaine aldehyde, and the betaine aldehyde produced was oxidized to betaine by BetB (betaine-aldehyde dehydrogenase) (Lamark et al., 1991). The *betA* and *betB* genes were found in the genomes of 252 and 251 *Acinetobacter* strains, respectively. The *otsB* gene, which is responsible for converting trehalose-6-P to trehalose, was found in the genomes of 230 *Acinetobacter* strains, while the *otsA* gene, which is responsible for the conversion of UDP-glucose to trehalose-6-P, was found in the genomes of 242 *Acinetobacter* strains (Figure 5). The *mtlD* gene, which encodes mannitol-1-phosphate dehydrogenase/phosphatase (MtlD), which is a key enzyme involved in mannitol biosynthesis (Zeidler et al., 2018), was found in 254 strains. The genes involved in the biosynthesis of betaine, trehalose, and mannitol were primarily found in clinical strains, particularly *A. baumannii*. Except *A. baumannii*, most clinical strains can synthesize two or three compatible solutes, and strains isolated from other environments can typically only synthesize one or two of the compatible solutes. No genes encoding for the synthesis of compatible solutes was found in 15 strains, which were isolated from animal feces (2), animal meat (1), water (5), swamp (2), soil (1), and creek mud (4). The biosynthesis of proline is usually catalyzed by glutamate 5-kinase (ProB), glutamate-5-semialdehyde dehydrogenase (ProA), and pyrroline-5-carboxylic acid reductase (ProC), while the synthesis of glutamate can be catalyzed by glutamate dehydrogenase (GdhA) or by a combination of glutamine synthetase (GlnA) and glutamate synthase (GltBD). All 312 strains can synthesize proline and glutamate to maintain the balance of cell osmotic pressure.

In this study, *A. pittii*, *A. calcoaceticus*, *A. radioresistens*, *A. terrae*, and *A. oleivorans* species had both clinical and environmental isolates. Comparative analysis revealed that these strains from the different isolation source shared the same type of potassium transport system and could synthesize the same compatible solutes. However, *A. lwoffii*, *A. junii*, and *A. soli* species contained different numbers of potassium ion transport system and compatible solute genes, but the clinical isolates contain more genes than the other environmental isolates, which may be due to the difference of osmotic pressure in isolated sources. The results showed that all *Acinetobacter* strains have different types of potassium ion transport systems and can synthesize some compatible solutes such as betaine, alginate, mannitol, proline, and glutamate, and these abilities enable the strains of *Acinetobacter* to more effectively adapt to the osmotic stress found in different environments.

### Defense against oxidative stress

**Figure 6:**
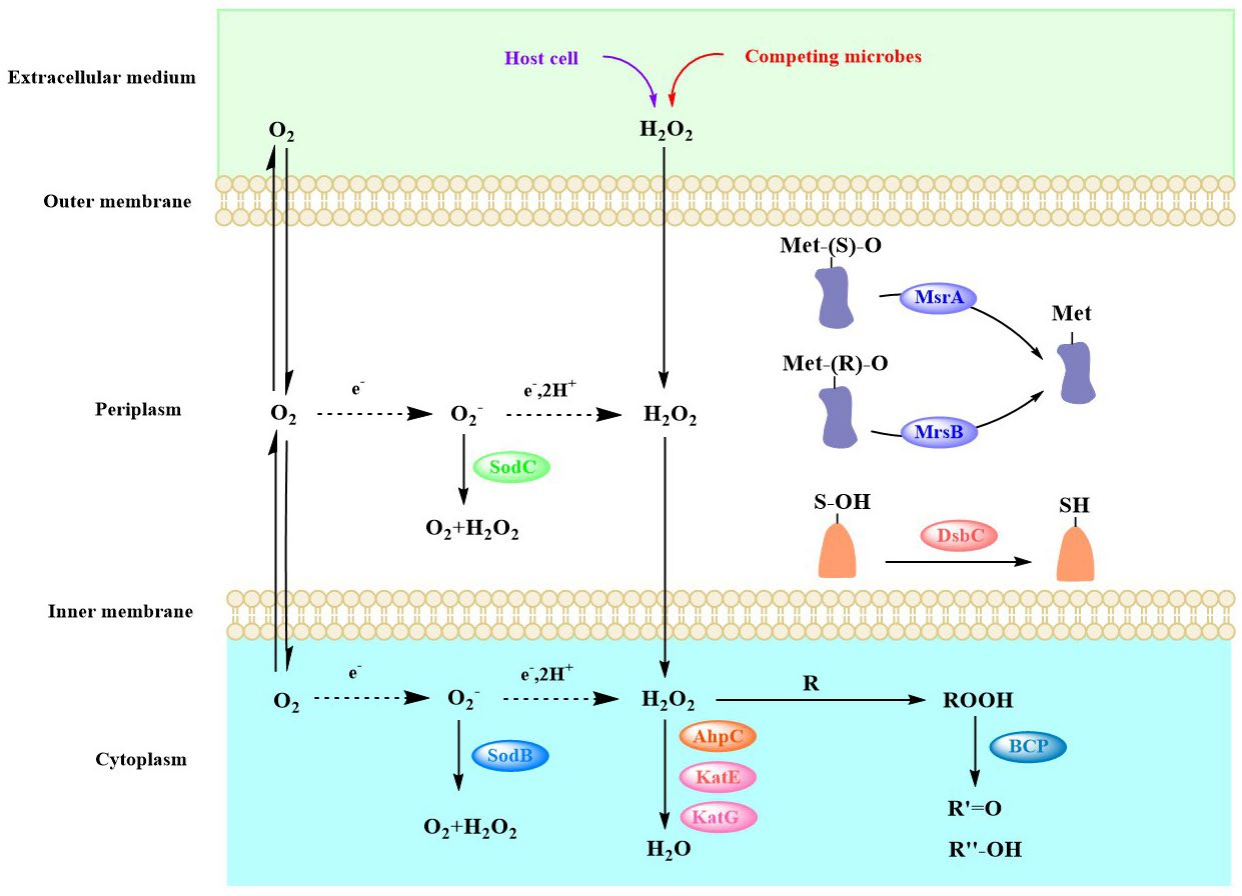
Various mechanisms that could be used by *Acinetobacter* to scavenge ROS and repair the membrane damage owing to ROS. ROS, reactive oxygen species.

A large amount of reactive oxygen species (ROS), such as hydrogen peroxide (H_2_O_2_), superoxide anion (O_2_^•-^), and hydroxyl radical (OH•), are produced in cells when the strains encounter extreme environmental conditions, such as drought (Noctor et al., 2014), high and low temperatures (Miteva-Staleva et al., 2014; Nantapong et al., 2019), high salinity (Song et al., 2018), low pH (Guo et al., 2016), and high loads of heavy metals (Neira et al., 2021). The massive accumulation of ROS in cells can kill the bacteria. Bacteria have evolved two primary mechanisms to protect themselves from the high concentration of ROS in their cells. One is to use enzymes to scavenge the ROS before they can cause much damage, and another is to repair the damaged biomolecules (Figure 6; Arts et al., 2015).

Superoxide dismutase (SOD) and catalase are the most common enzyme to remove the ROS in bacterial cells. SOD catalyzes the dismutation of O_2_^•−^ to O_2_ and H_2_O_2_, and since O_2_^•−^ does not easily diffuse through biofilms, varying types of SODs exist in different cellular compartments (Figure 6). For example, in *Acinetobacter* sp. Ver3, SodB is a cytoplasmic enzyme, while SodC is found in the periplasm (Steimbruch et al., 2022). The gene *sodC* was found in the genomes of most *Acinetobacter* strains (298 genomes). Although the *sodC* gene was not found in the other 14 strains, these strains could produce SodB, which strains can use to convert O_2_^•−^ -to O_2_ and H_2_O_2_ to destroy the superoxide anion. In most organisms, catalase (KatE and KatG) and alkylhydroperoxidase (AhpC) catalyze the conversion of H_2_O_2_ (Imlay, 2013). The *ahpC* gene was present in all the genomes of these *Acinetobacter* strains. The *katE* gene was present in 297 *Acinetobacter* strains, and the *katG* gene was present in 223 strains. In particular, *katG* was primarily present in *A. baumannii* (Figure 5). The peroxiredoxin Bcp, which reduces a wide range of substrates but is catalytically inefficient (Jeong et al., 2000), is present in all 312 *Acinetobacter* strains.

Cysteine and methionine residues, which are found in many proteins, are easily oxidized by the ROS to sulfonic acid derivatives (-SOH) and methionine sulfoxide (Met-O), respectively, which results in the inactivation, misfolding, and even degradation of enzymes (Davies, 2005; Roos and Messens, 2011). DsbA introduces disulfide bonds between consecutive cysteine residues, which leads to potential mismatches. The *dsbC* gene that encodes the disulfide isomerase DsbC was found in all 312 *Acinetobacter* strains, which enables them to correct this mismatch and repair the oxidized cysteine residues (Darby et al., 1998). The methionine sulfoxide reductase (Msr) system reduces Met-O residues to methionine, with MsrA specifically reducing S-Met-O, while MsrB preferentially reduces the R-Met-O isomer (Grimaud et al., 2001). The *msrA* gene was found in all 312 strains of *Acinetobacter*, while the *msrB* gene was found in 310 strains; it was only absent from the genomes of *A. geminorum* J00019^T^ and *A. beijerinckii* CIP 110307 that were isolated from clinical sources (Figure 5). The results of the genome analysis revealed that the *Acinetobacter* strains have different ways to remove the ROS and repair ROS damage, which allows the strains to easily face the accumulation of ROS under extreme environmental conditions and enhance environmental adaptability.

### Analysis of the resistome of the *Acinetobacter* strains

Antimicrobial compounds released into the environment from households, hospitals, pharmaceutical systems, and agricultural runoff come into direct contact with natural bacterial communities, which will cause bacteria to be killed by antibiotics and also contribute to the proliferation of more antibiotic-resistant bacteria (Lin et al., 2021). Thus, the presence of antibiotic resistance genes provides for strains to cope with antibiotics in the environment. In addition, the antibiotic resistance genes (ARGs) can lead to an increase in the ARG pools in environmental bacteria and facilitate the transfer of resistance to existing and emerging pathogens, which seriously endangers human health (Czekalski et al., 2012).

A total of 36 antibiotic efflux genes, 222 antibiotic inactivation genes, 6 antibiotic target alteration genes, 3 antibiotic target protection genes, and 8 antibiotic target replacement genes were found in all these *Acinetobacter* strains (Supplementary Table S4). The least number of ARGs was identified in *A. baretiae* B10A^T^ with four, and the largest number was *A. baumannii* TG2013, which had 47. The number of ARGs contained in clinical strains ranged from 7 to 47 (average of 28 per strain), with *A. baumannii* contained number of ARGs from 13 to 47 and the rest of clinical strains from 7 to 38. In addition, the number of ARGs ranged from 4 to 27 (average 12 per strain) for animal strains and from 5 to 28 (average 12 per strain) for environmental strains. It revealed that the ARGs number of clinical strains is higher than that in the other environments, which may be related to the isolated environment with higher antibiotic pressure. For *A. pittii* species, there was no difference in the number of ARGs for clinical and environmental isolates, which ranged from 21 to 22 for clinical isolates and 22–24 for environmental isolates. Similarly, for *A. radioresistens*, *A. calcoaceticus*, *A. radioresistens*, *A. junii*, *A. oleivorans*, *A. soli* species, there was no difference in ARGs between the clinical isolates and the other environmental isolates.

The *rsmA* and *parC* genes that are related to adhesion and quinolone resistance, respectively, were detected in all 312 strains (Nowroozi et al., 2014; Sun et al., 2020). The AdeIJK efflux pump gene cluster was detected in 293 *Acinetobacter* strains that developed resistance to various drugs, including β-lactams, such as ticarcillin, cephalosporins, aztreonam, fluoroquinolones, and tetracyclines among others (Rosenfeld et al., 2012), while the *adeFGH* efflux pump was detected in the genomes of 189 *Acinetobacter* strains. The OXA β-lactamase gene was detected in 183 clinical strains, 15 animal strains and 38 environmental strains, and it was only absent in *A. baumannii* 1042969-1265 and *A. baumannii* 348935 among the 145 strains of *A. baumannii*. In particular, OXA-23 and OXA-66 that encode a β-lactamase variant that is resistant to penems, carbapenems, and cephalosporins, was found in 48 strains, including 44 of *A. baumannii*, and 33, including 31 of *A. baumannii*, respectively. Among them, 15 strains simultaneously possessed OXA-23 and OXA-66. The highest number of antibiotics associated with the resistance mechanisms were cephalosporins, penems, and carbapenems, with 174, 109, and 104 resistance proteins, respectively. In contrast, there were low amounts of antimicrobial compounds, such as sulfonamide, fosfomycin, cephamycin, and monobactam, and subsequently low amounts of resistance mechanisms related to the resistome were predicted for *Acinetobacter*. Therefore, the data obtained in this study can be used to develop new drug candidates and use the assembled pan-resistance to predict the possible mechanisms that *Acinetobacter* can utilize to prevent its eradication.

### Analysis of *Acinetobacter* mobile genetic elements

Genomics methods can not only analyze the gene structure and evolutionary relationship between microbial genomes but also analyze the HGT between bacterial genomes. HGT is one of the efficient adaptation strategies for strains in adverse environments, and extensive gene recruitment *via* HGT expands genomic diversity within the species (Zhang et al., 2018). Mobile genetic element is a specific genome segment, which is regarded as a sign of HGT events, including phage and GIs (Ullrich et al., 2016). Temperate phages are important factors in bacterial evolution and the formation of new pathogens, as well as important enablers of gene flow (Hendrix, 2003; Hanlon, 2007). Phages promote the formation of GIs and serve as mobile genetic components that confer superior mobility through site-specific recombination and integration into the corresponding chromosomes (Osborn and Boltner, 2002). A total of 1,353 prophages were identified from these 312 *Acinetobacter* strains, including 299 intact (completeness score > 90) prophages, 200 questionable (completeness score of 60–90) prophages, and 854 incomplete (completeness score < 60) prophages (Supplementary Table S5). Among them, *Acinetobacter* sp. 243_ASPC had the highest number of prophages (12), while *A. baumannii* AKBS16, *A. lactucae* NRRL B-41902^T^, *A. pittii* PHEA-2, *Acinetobacter* sp. AR2-3 and *A. nosocomialis* M2 had no detected prophage regions. The numbers of prophages in the other strains ranged from 1 to 11 (Supplementary Table S1).

The GIs contained clusters of genes that were acquired as a single unit by HGT. It can increase the versatility of recipient bacteria and enable the host bacteria to adapt to highly diverse ecological niches. The analysis showed that each strain of *Acinetobacter* contained multiple GIs, which ranged in number from 6 to 70 (Supplementary Table S1). The strains with the least number of GIs were *A. calcoaceticus* RUH2202, *A. oleivorans* NIPH 542, and *A. baylyi* DSM 14961 (6), while *A. idrijaensis* MII harbored the most GIs (70). The GIs are responsible for the unique aspects of bacterial behavior, and each of these important genes brings new possibilities to this population (Waterfield et al., 2002). Some Gls have genes related to the resistance of aminoglycoside antibiotics (98 strains), β-lactamase types (101 strains), macrolide antibiotics (28 strains), tetracycline (58 strains), nucleotidyl transferase (62 strains), and multidrug efflux pumps (46 strains; Supplementary Table S6). The multitude of data and functions of GIs indicate that mobile elements could be involved in the pathogenicity and drug resistance of the strains, as well as interactions with the host, during the process of evolution of the population. Therefore, the prevalence of prophage and GIs in the *Acinetobacter* genomes may imply rapid adaptation occurred to give them a survival advantage in diverse environments.

### HGT analysis of alkane and aromatic hydrocarbon genes

According to the phylogenetic tree based on the core genome (Figure 1A), AlkM (Figure 1B) and AlmA (Figure 1C), these *Acinetobacter* strains could be assigned into 11, 12 and 11 clusters, respectively. The topology of the AlkM sequence-based phylogenetic tree is similar to that of the core genome-based phylogenetic tree, with clusters I, II, III, IV, V, IX, and X all clustered together. However, there are some minor differences. For example, *Acinetobacter* sp. YH16032, which belongs to cluster VIII in the core genome, is clustered with cluster I in the AlkM phylogenetic tree. *A. marinus* ANC 3699 of clusters IV and VI in the core phylogenetic tree clustered together with cluster XII in the AlkM phylogenetic tree. For strains that contain two or three copies of the *alkM* gene, one AlkM sequence was distributed in clusters III, IV, VII and VIII, while the other AlkM sequences were distributed in cluster XII. Similarly, the topology of the AlmA sequence-based phylogenetic tree was also similar to that based on the core genome with only minor differences. In the core phylogenetic tree, *A. pullicarnis* S23^T^ belonged to cluster VII, and *Acinetobacter* sp. YH16032 and *A. shaoyimingii* 323-1^T^ belonged to cluster VIII, which were inserted into cluster I in the AlmA phylogenetic tree. In addition, clusters IX and X in the core phylogenetic tree also clustered with cluster I in the AlmA phylogenetic tree. Alternatively, the topology of the phylogenetic tree based on the sequences of CatA (Figure 1D), BenA (Figure 1E), and BenB (Figure 1F) differed significantly from that based on the core genome. Strains that clustered in one cluster in the core genome phylogenetic tree were usually scattered in different branches in the CatA, BenA and BenB phylogenetic trees.

The G + C content of the 312 *Acinetobacter* genomes was 34.9–45.4%. The G + C content of the *alkM*, *almA*, and *benB* genes was similar to the host genome, while that of the *catA* and *benA* genes differed from the host genome. The G + C contents of *alkM*, *almA* and *benB* genes were 35.9–46.7%, 36.6–46.4%, and 34.6–46.1%, respectively, while the G + C contents of *catA* and *benA* genes were 40.0–53.9% and 39.4–47.4%, respectively (Supplementary Figure S3). In addition, the *alkM*, *almA*, *catA*, *benA* and *benB* genes were not found in the GIs and prophage regions.

## Discussion

One of the main aspects related to habitat adaptation is nutrient availability and another is resistance to extreme environment. The *Acinetobacter* strains can survive in diverse ecological environments with different nutrients, osmotic pressure, oxidative conditions, and the presence of antibiotics. These factors can make their living conditions far from optimal. Therefore, the *Acinetobacter* strains need to possess multiple strategies to cope with this type of environment.

Carbon and energy resources are a key limiting factor for the survival of bacteria in the environment. A comparative genomic analysis showed that most of the *Acinetobacter* strains harbored the *alkB*/*alkM* and *almA* genes, as well as genes related to the degradation of fatty acids, suggesting that they can utilize medium-and long-chain alkanes and fatty acids (the metabolites of alkanes) as the carbon and energy sources for their growth. Previous studies had proven that many *Acinetobacter* strains could degrade *n*-alkane as the sole carbon and energy source for their growth (Sun et al., 2012). The hydrocarbons can be produced through the fatty acid metabolism pathways of plants (Samuels et al., 2008), microorganisms (Lea-Smith et al., 2015), insects (Tillman et al., 1999), and humans (Biedermann et al., 2015). In addition, hydrocarbons are the primary components of crude oil (Varjani, 2017). The wide distribution of the hydrocarbon compounds in the environment means that the *Acinetobacter* strains can easily take them up from the environment. More importantly, fatty acids are the metabolites produced during the degradation of hydrocarbons and can also be used as their carbon source. The genome analysis showed that the *Acinetobacter* strains could utilize the fatty acids generated by the acyl CoA and β-oxidation pathways. Most of the *Acinetobacter* had alkanes and aromatic hydrocarbons degradation genes, which indicated that *Acinetobacter* could be applied to bioremediation of petroleum contaminated sites. A small portion of the *Acinetobacter* strains did not harbor the *alkM*/*alkB* and *almA* genes. This could be attributed to the incomplete genome sequence or gene loss during evolution to adapt to the environment. Furthermore, these *Acinetobacter* strains harbored the intact gluconeogenesis pathway, citric acid cycle (TCA cycle), pentose phosphate pathway, and oxidative phosphorylation. Although *Acinetobacter* strains lack glucose phosphokinase, they cannot utilize glucose by themselves. In contrast, they can easily obtain glucose-1-phosphate from the host (human, animal, or plant) or the other bacteria in the same niches by substance exchange. For example, *Agrobacterium tumefaciens* can obtain glucose-1-phosphate from its environment by active transport (Fukui and Miyairi, 1970). The comparative genomic analysis suggests that as non-motile chemoorganotrophs, the *Acinetobacter* strains can easily obtain their carbon and energy sources from the environment. In addition to their high abilities to take up carbon resources, *Acinetobacter* commonly contains siderophores, which enable the strains to efficiently take up iron from the environment. Strains can create a competitive environment by acquiring iron from their habitat and causing lethal effects on their competing microbes from iron deficiency (Niehus et al., 2017). However, not all of the strains contained the genes for the biosynthesis of siderophores, possibly because of their ecological dependence on other siderophore producers or the presence of new types of siderophores (Kramer et al., 2020).

The survivability of bacteria in the environments also relied on their abilities to synthesize primary and secondary metabolite by themselves. The results of a comparative analysis revealed that the *Acinetobacter* strains were able to independently synthesize purines, pyrimidines, fatty acids, peptidoglycans, and most of the amino acids. Moreover, the *Acinetobacter* strains could also biosynthesize various secondary metabolites, such as arylpolyene, β-lactone, and siderophores. In this study, there were many smBGCs with a low percentage of similarity, which could indicate that each cluster has novel secondary metabolites. In addition, the presence or absence of specific gene clusters in the strains studied can explain their adaptation to specific habitats. We can use the data obtained in this work to design an enrichment medium to improve the isolation efficiency of *Acinetobacter* from various environment, and this will be an interesting topic for future work.

Extreme conditions, including osmotic pressure, oxidative conditions, and antibiotics among others, is another limiting factor for the survival of strains in the environment. Thus, *Acinetobacter* strains possess a series of osmotic pressure protection, anti-oxidative stress, and antibiotic resistance genes to protect them from extreme environmental stress. Compatible solutes are the main strategy of the strain to cope with the high-osmolality environment. Previous studies revealed that mannitol and glutamic acid are the primary compatible solutes of *A. baumannii* (Zeidler et al., 2017; Zeidler and Müller, 2019), whereas betaine and trehalose were also found to be important compatible solutes in *A. baumannii* in this study. Compared with the strains isolated from other environments, the pathogenic bacteria usually have more genes that are compatible for the synthesis of solutes, which could be owing to the high osmotic pressure in the environment where pathogenic bacteria exist, such as blood, intestinal lumen, and urine (Chowdhury et al., 1996; Culham et al., 2001). In addition, many strains in the natural environment are not affected by hypertonicity and desiccation, and the bacterial uptake of compatible solutes is superior to their *de novo* synthesis, which could result in some environmental strains having few genes related to the synthesis of compatible solutes (Oren, 1999). Glutamate and proline as amino acids can be commonly synthesized in *Acinetobacter*, so they can be used as the primary compatible solutes regardless of the high or low osmotic pressure of the isolated environment. This suggests that the *Acinetobacter* strains have different strategies to cope with the osmotic pressure environment. Under lower external NaCl conditions, the *Acinetobacter* strains were primarily able to adapt to their environment through the potassium ion transport system, glutamate, and proline, while under high NaCl conditions, more compatible solutes need to be synthesized.

ROS are accumulated in large amounts by strains in extreme environments, and they can originate from the host immune system and other microbial and abiotic sources. High levels of ROS can damage bacterial DNA and membrane lipids and proteins, which are deleterious to their survival. Therefore, in response to ROS stress from the external environment, the *Acinetobacter* strains can rapidly scavenge excess reactive oxygen species by SODs and catalase, as well as by promptly repairing damaged cysteine residues and methionine residues in the envelope, using the DsbC and Msr systems, respectively. These enzymes are ubiquitous in *Acinetobacter*, which allows it to be protected from the stress of highly reactive oxygen species in a variety of extreme environments and ensures its normal membrane function. Normal membrane function is essential for the survival of strains and the integrity and synthesis of peptidoglycan.

The intricate link between humans, animals and their environment has led to antibiotics being common in the natural environment (Allen et al., 2010). In response to this environmental pressure, strains need to possess antibiotic resistance genes, which may increase the incidence of multidrug resistance. Efflux pump and antibiotic resistance genes were detected in all *Acinetobacter* strains regardless of the source of their isolation. The efflux pump gene is a key factor that mediates the resistance of bacteria to antibiotics, and these genes play roles not only in antibiotic resistance under high antibiotic selection pressure but also in intercellular communication, cellular detoxification, and bacterial homeostasis in natural ecosystems under low antibiotic pressure (Piddock, 2006). Previous studies have shown that the most common efflux pumps in *Acinetobacter* are AdeABC, AdeFGH and AdeIJK, which is consistent with our results (Baraka et al., 2021). In addition, *Acinetobacter* contains several types of β-lactamases, including OXA-23-like, OXA-66-like, and OXA-82-like, which could render them resistant to broad-spectrum β-lactam antibiotics, particularly penems, carbapenems and cephalosporins. These efflux pump and antibiotic resistance genes increase the ability of *Acinetobacter* to survive in an environment that contains high levels of antibiotics and makes the strains of *Acinetobacter* naturally resistant to many antibiotics. It is noteworthy that *Acinetobacter* was found to be generally resistant to the β-lactam antibiotics in our study, and therefore the use of these drugs in *Acinetobacter* is contraindicated. However, we can use this pan-resistance to investigate the mechanisms that *Acinetobacter* may use to prevent its eradication. In addition, *Acinetobacter* contained fewer resistance genes associated with sulfonamide, fosfomycin, cephamycin and monobactam antibiotics, which could be used to some extent in the selection and development of drug candidates. In summary, the genes related to osmotic protection, antioxidative stress, and antibiotic resistance enhance the viability of *Acinetobacter* strains under extreme conditions in the environment or host.

HGT can contribute to the variability in the contents of prokaryotic genome, which is closely related to microbial adaptation and evolution. HGT events with beneficial effects increase receptor fitness and eventually become fixed in the population, while redundant or deleterious acquired genes are subject to selection and accumulate mutations before being lost (Treangen and Rocha, 2011). The phylogenetic tree based on alkane-degrading AlkM and AlmA sequences differed slightly from the core genome phylogenetic tree in topology, and the *alkM* and *almA* genes were less distinct from the genome in terms of G + C content. In contrast to the alkane-degrading genes, the phylogenetic tree based on CatA, BenA, and BenB differed significantly from the core genome phylogenetic tree in topology. The G + C content of the *catA* and *benA* genes differed significantly from that of the genome, while the *benB* gene was similar to that of the genome. The sequences of *alkM*, *almA*, *catA*, *benA* and *benB* genes were not found in the GIs and prophage, which could be owing to some defects in the accuracy of predicting GIs and prophages. Therefore, the analysis could not reveal an obvious recent HGT event of *alkM* and *almA* genes in *Acinetobacter*, but the *catA*, *benA*, and *benB* genes could have been acquired by HGT. In addition, *Acinetobacter* can also expand its gene pool through various mobile elements. Different amounts of horizontally transferred GIs and prophages were found in each *Acinetobacter* genome, and the genes related to multidrug efflux pumps and antibiotic resistance were found in the GIs, which once again suggests that HGT is a major factor in the emergence of antibiotic resistance genes and could play an important role in the evolution of *Acinetobacter* (Abdi et al., 2020). The open pan-genome and growing number of new genes also illustrate that the *Acinetobacter* strains are able to introduce alien genes through genetic exchange with other community members in common microhabitats. Collectively, gain and loss of genes or horizontal exchange of abundant genetic material are also efficient adaptation strategies of *Acinetobacter* strains in these adverse environments.

## Conclusion

The genome analysis of 312 *Acinetobacter* strains shows that they have a strong metabolic capacity and can utilize medium-and long-chain alkanes and aromatic hydrocarbons, such as catechol and benzoic acid, in ubiquitous environments as carbon and energy sources. In addition, to resist and adapt to external environmental stress, the genomes of all the strains accumulated many functional genes related to antioxidative stress, protection from hyperosmotic pressure, antibiotic resistance, and secondary metabolite synthesis. *Acinetobacter* can also accept foreign genes through mobile elements, such as GIs and prophages, in which the *alkM* and *almA* genes for alkane degradation may not be acquired by HGT, while *catA*, *benA*, *benB* and antibiotic resistance genes may be acquired by HGT. The genomic features of this study will enable a better understanding of how *Acinetobacter* adapts to various ecological niches.

## Data availability statement

The original contributions presented in the study are included in the article/Supplementary material, further inquiries can be directed to the corresponding author.

## Author contributions

J-QS and YZ designed the work, analyzed the data, and wrote the manuscript. YZ, H-MW, J-LY, and LX collated the data. J-QS guided the data analysis and revised the manuscript. All authors contributed to the study and approved the final submitted version.

## Funding

This work was supported in part by National Natural Science Foundation of China (Grant Nos. 31960020 and 32260022) and High-Level Talent Start-Up Research Project of Inner Mongolia University (No. 21800–5185133).

## Conflict of interest

The authors declare that the research was conducted in the absence of any commercial or financial relationships that could be construed as a potential conflict of interest.

## Publisher’s note

All claims expressed in this article are solely those of the authors and do not necessarily represent those of their affiliated organizations, or those of the publisher, the editors and the reviewers. Any product that may be evaluated in this article, or claim that may be made by its manufacturer, is not guaranteed or endorsed by the publisher.

## Footnotes

^1^http://jspecies.ribohost.com/jspeciesws/

^2^http://ggdc.dsmz.de/ggdc.php

^3^https://blast.ncbi.nlm.nih.gov/Blast.cgi

^4^https://itol.embl.de/

^5^https://antismash.secondarymetabolites.org/

^6^https://card.mcmaster.ca/

^7^http://phaster.ca/

^8^http://www.pathogenomics.sfu.ca/islandviewer/

## Supplementary material

SUPPLEMENTARY TABLE S1 Basic Information of 312 Acinetobacter strains.

SUPPLEMENTARY TABLE S2 Genes predicted to be involved in the central metabolism, nitrogen, phosphte, sulphate and energy metabolism in the Acinetobacter strains.

SUPPLEMENTARY TABLE S3 Prediction of 21 Acinetobacter complete genome secondary metabolite biosynthetic gene clusters.

SUPPLEMENTARY TABLE S4 Prediction of antibiotic resistance genes in 312 Acinetobacter strains.

SUPPLEMENTARY TABLE S5 Predicted Phage proteins in Acinetobacter spp. using PHASTER.

SUPPLEMENTARY TABLE S6 The significant functional genes in GIs of Acinetobacter.

SUPPLEMENTARY FIGURE S1 COG distribution of 312 Acinetobacter strains. **(A)** COG distribution of core genes, accessory genes and unique genes in information storage and processing, cellular processes and signaling, metabolism and poorly characterized. **(B)** COG distribution of core, accessory and unique genes. Classes D, M, N, O, T, U and V are involved in Cellular processing and signaling. Classes J, K, and L are involved Information storage and processing. Classes C, G, E, F, H, I, Q and P are involved in Metabolism. Classes R and S belong to the category of Poorly characterized.

SUPPLEMENTARY FIGURE S2 COG distribution of 33 typic Acinetobacter strains. **(A)** COG distribution of core genes, accessory genes and unique genes in information storage and processing, cellular processes and signaling, metabolism and poorly characterized. **(B)** COG distribution of core, accessory and unique genes. Classes D, M, N, O, T, U and V are involved in Cellular processing and signaling. Classes J, K, and L are involved Information storage and processing. Classes C, G, E, F, H, I, Q and P are involved in Metabolism. Classes R and S belong to the category of Poorly characterized.

SUPPLEMENTARY FIGURE S3 Comparison of alkM, almA, catA, benA and benB genes with the G+C content of strain genome. **(A)** alkM gene and genome. **(B)** almA gene and genome. **(C)** catA gene and genome. **(D)** benA gene and genome. **(E)** benB gene and genome.
