# Supplementary figures and images for: A comprehensive genomic analysis provides insights on the high environmental adaptability of Acinetobacter strains
Source: Front Microbiol. 2023 Apr 17;14:1177951. doi: 10.3389/fmicb.2023.1177951 (PMC10149724; doi:10.3389/fmicb.2023.1177951)

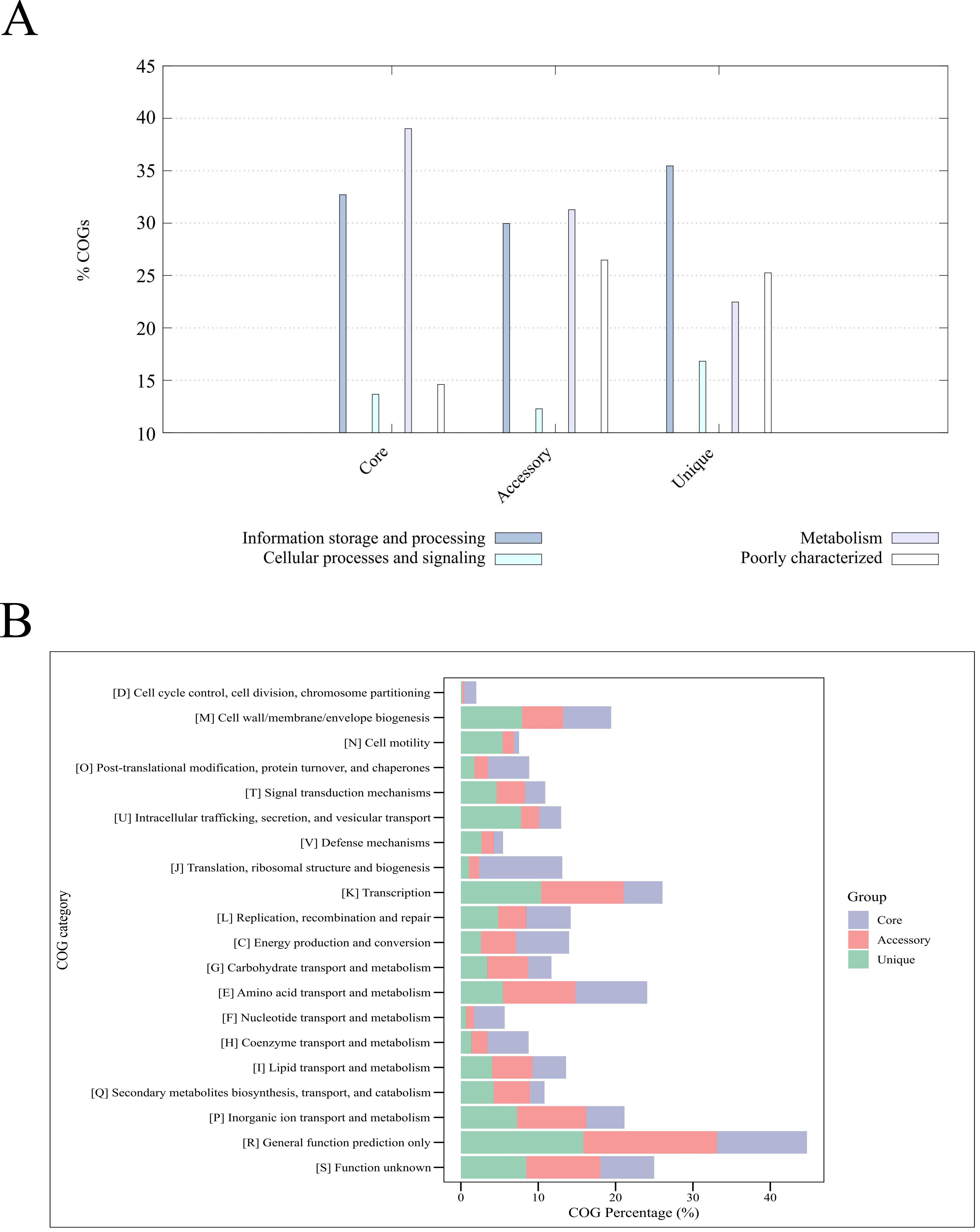

Supplement: SUPPLEMENTARY FIGURE S1 — COG distribution of 312 Acinetobacter strains. (A) COG distribution of core genes, accessory genes and unique genes in information storage and processing, cellular processes and signaling, metabolism and poorly characterized. (B) COG distribution of core, accessory and unique genes. Classes D, M, N, O, T, U and V are involved in Cellular processing and signaling. Classes J, K, and L are involved Information storage and processing. Classes C, G, E, F, H, I, Q and P are involved in Metabolism. Classes R and S belong to the category of Poorly characterized. [file Image_1.JPEG]

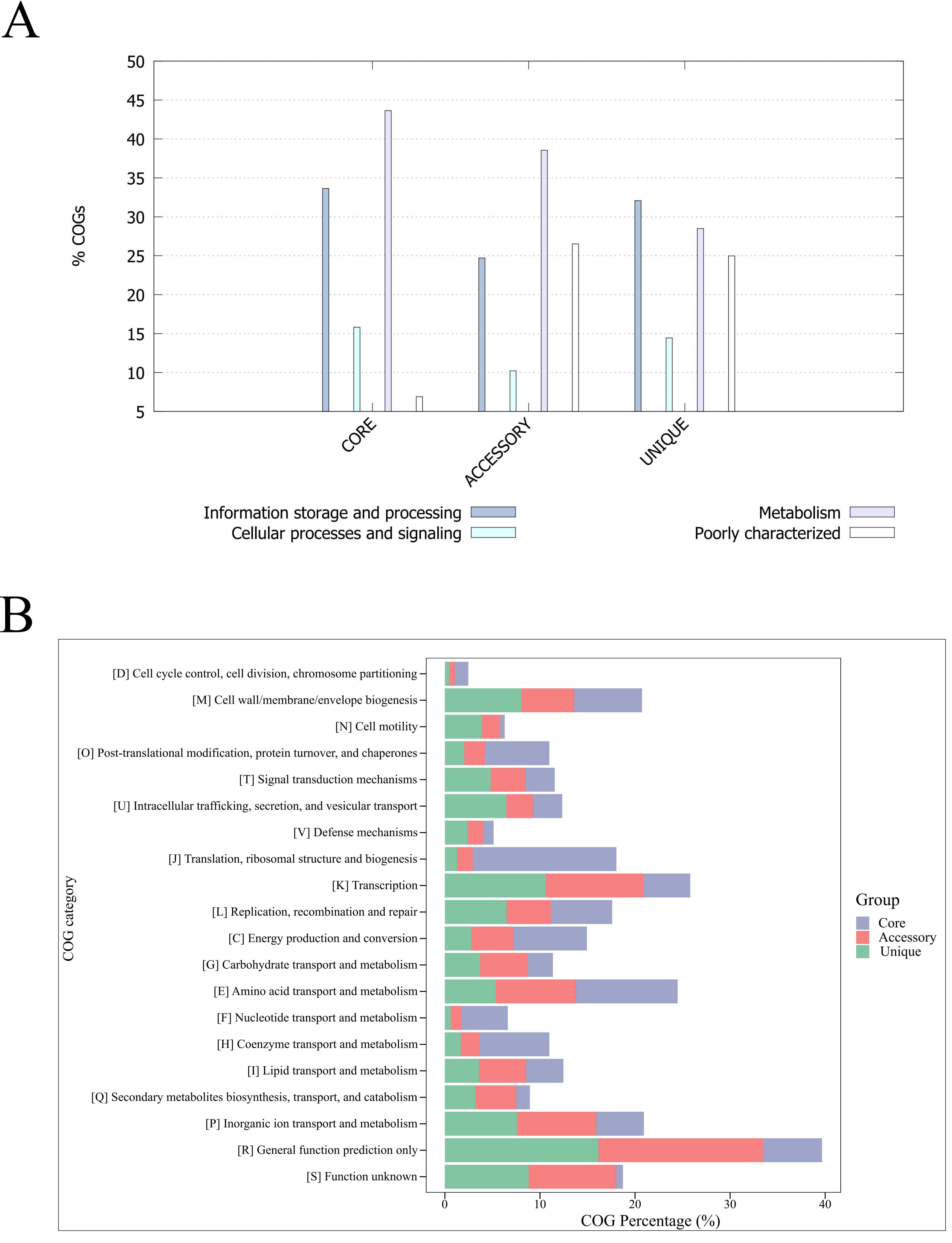

Supplement: SUPPLEMENTARY FIGURE S2 — COG distribution of 33 typic Acinetobacter strains. (A) COG distribution of core genes, accessory genes and unique genes in information storage and processing, cellular processes and signaling, metabolism and poorly characterized. (B) COG distribution of core, accessory and unique genes. Classes D, M, N, O, T, U and V are involved in Cellular processing and signaling. Classes J, K, and L are involved Information storage and processing. Classes C, G, E, F, H, I, Q and P are involved in Metabolism. Classes R and S belong to the category of Poorly characterized. [file Image_2.JPEG]

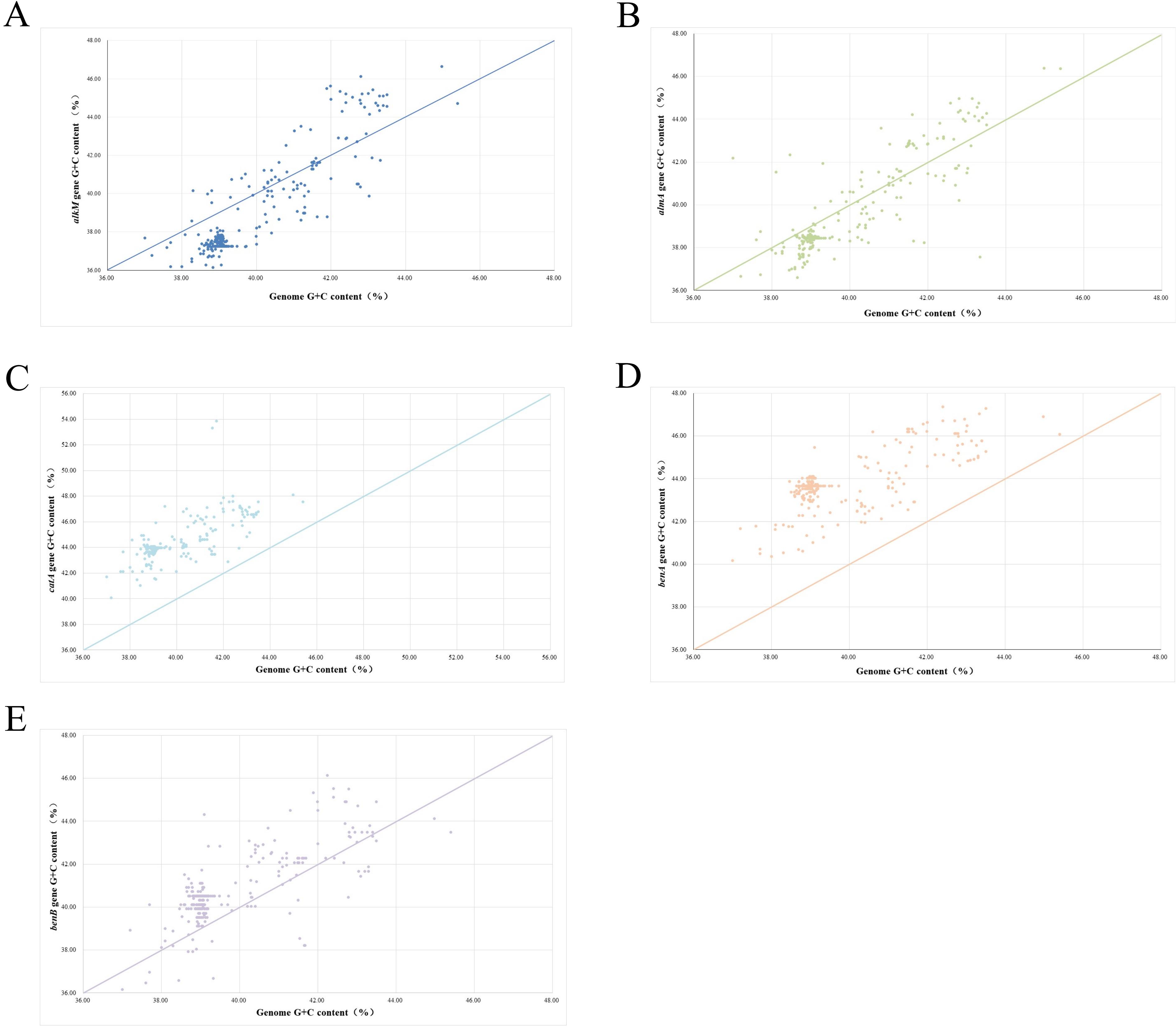

Supplement: SUPPLEMENTARY FIGURE S3 — Comparison of alkM, almA, catA, benA and benB genes with the G+C content of strain genome. (A) alkM gene and genome. (B) almA gene and genome. (C) catA gene and genome. (D) benA gene and genome. (E) benB gene and genome. [file Image_3.JPEG]
